# Speaking up for patient safety by hospital-based health care professionals: a literature review

**DOI:** 10.1186/1472-6963-14-61

**Published:** 2014-02-08

**Authors:** Ayako Okuyama, Cordula Wagner, Bart Bijnen

**Affiliations:** 1 Department of Total Health Promotion Science, School of Health Sciences, Graduate School of Medicine, Osaka University, Yamadaoka 1-7, Suita-shi 565-0871, Osaka, Japan; 2 VU University Medical Center, van der Boechorststraat 7, 1081 BT Amsterdam, The Netherlands; 3 NIVEL Netherlands Institute for Health Services Research, P.O. Box 1568, 3500 BN Utrecht, The Netherlands; 4 Foreest Medical School, Medical Centre Alkmaar, Wilhelminalaan 12, 1815 JD Alkmaar, The Netherlands

**Keywords:** Speaking up, Inter-professional relations, Patient care team, Patient safety, Communication

## Abstract

**Background:**

Speaking up is important for patient safety, but often, health care professionals hesitate to voice concerns. Understanding the influencing factors can help to improve speaking-up behaviour and team communication. This review focused on health care professionals’ speaking-up behaviour for patient safety and aimed at (1) assessing the effectiveness of speaking up, (2) evaluating the effectiveness of speaking-up training, (3) identifying the factors influencing speaking-up behaviour, and (4) developing a model for speaking-up behaviour.

**Methods:**

Five databases (PubMed, MEDLINE, CINAHL, Web of Science, and the Cochrane Library) were searched for English articles describing health care professionals’ speaking-up behaviour as well as those evaluating the relationship between speaking up and patient safety. Influencing factors were identified and then integrated into a model of voicing behaviour.

**Results:**

In total, 26 studies were identified in 27 articles. Some indicated that hesitancy to speak up can be an important contributing factor in communication errors and that training can improve speaking-up behaviour. Many influencing factors were found: (1) the motivation to speak up, such as the perceived risk for patients, and the ambiguity or clarity of the clinical situation; (2) contextual factors, such as hospital administrative support, interdisciplinary policy-making, team work and relationship between other team members, and attitude of leaders/superiors; (3) individual factors, such as job satisfaction, responsibility toward patients, responsibility as professionals, confidence based on experience, communication skills, and educational background; (4) the perceived efficacy of speaking up, such as lack of impact and personal control; (5) the perceived safety of speaking up, such as fear for the responses of others and conflict and concerns over appearing incompetent; and (6) tactics and targets, such as collecting facts, showing positive intent, and selecting the person who has spoken up.

**Conclusions:**

Hesitancy to speak up can be an important contributing factor to communication errors. Our model helps us to understand how health care professionals think about voicing their concerns. Further research is required to investigate the relative importance of different factors.

## Background

Learning effective communication and teamwork skills is crucial to improving patient safety for health care professionals [1]. The frontline staff, such as medical residents and nurses, is well positioned to observe early signs of unsafe conditions in care delivery and bring them to the attention of the organisation [2,3]. ‘Speaking up’ is defined as the raising of concerns by health care professionals for the benefit of patient safety and care quality upon recognising or becoming aware of the risky or deficient actions of others within health care teams in a hospital environment [4,5]. Such actions include mistakes (e.g. missed diagnoses, poor clinical judgement), lapses, rule breaking, and failure to follow standardised protocols. Speaking up is expected to have an immediate preventive effect on human errors or to improve technical and system deficiencies. Organisational research illustrates that, in many cases, people choose the ‘safe’ response of silence, withholding input that could be valuable to others or thoughts that they wish they could express [6,7]. In health care environments, it has been shown that those who are aware of a problem often either speak up and are ignored or do not speak up at all [8,9].

Previous organisational studies indicated that several factors influence employees’ voicing behaviour. Silence can be caused by fear, by the desire to avoid conveying bad news or unwelcome ideas, and by normative and social pressures that exist in groups [6,7]. In addition, hesitance in speaking up or failure to indicate or correct errors can be caused by disproportionate authority gradients, excessive professional courtesy, and/or deficiencies in resource or task management [10]. Morrison integrated the existing theory and research and developed the model of employee voice [11]. In this model, it is presumed that the driving motive for voice is the desire to help the organisation or work unit to perform more effectively or to make a positive difference for the collective. The voice reflects a deliberate decision process whereby the individual considers both positive and negative consequences and the perceived efficacy and safety of voicing his or her concerns. The perceived efficacy of voice is the individual’s judgement about whether it is likely to be effective. The perceived safety of voice is the individual’s judgement about the risk of potential negative outcomes. The individual is faced with a balancing act of trying to be pro-social and constructive while at the same time being mindful of personal costs. Contextual factors (e.g. organisational culture) and individual factors (e.g. job attitude, personality) affect these perceptions. The employee’s voice has important benefits for organisations and work groups as well as for the one who speaks up. The message type, tactic, and target are also important factors in voicing.

The Morrison model for organisations provides us with a basic framework, but for the clinical setting two factors have to be taken into account. The first is that the type of information that is being conveyed is usually one of concern [11]. An employee may for instance think very differently about the potential benefits and risks of speaking up when bringing up such an issue of concern compared to voicing a novel suggestion. The second is that while in organisational contexts speaking up will often relate to the well-being and goals of the organisation and its workers, speaking up in health care for patient safety is primarily aimed at promoting the well-being of its clients. In health care, several interventions have been introduced to improve teamwork and communication [12]. While teaching safety theory and/or team training may not be sufficient to empower health care professionals to voice their concerns [13], understanding speaking-up behaviour and its related factors can be useful in designing patient safety improvement initiatives that lead to more effective and sustainable behavioural change and safety improvement outcomes. This review was aimed at developing a model that integrates evidence from the existing literature on health care professionals’ speaking-up behaviour on the basis of their particular characteristics (e.g. concerns related to patients’ well-being). Such a model is expected to help us to understand why health care professionals often prefer silence to speaking up when patient safety is at stake. While there have been a growing number of studies on factors that enhance or inhibit speaking up by health care professionals recently, a conceptualised theoretical model for understanding speaking-up behaviour and its related factors is not yet available. In light of this, the current review aims at (1) assessing the effectiveness of speaking up for patient safety, (2) evaluating the effectiveness of speaking-up training, (3) identifying the influencing factors of speaking-up behaviour by health care professionals, and (4) developing a model for health care professionals’ speaking-up behaviour by integrating these factors into the model of employee speaking-up behaviour. This study does not consider whistle-blowing to the public or the authorities but focuses on performance monitoring within teams for patient safety. Likewise, our study focuses on the preventive aspect of speaking up rather than on other aspects such as sharing of ideas.

## Methods

### Data sources

Relevant English-language articles published up to and including December 2012 were sourced using PubMed, MEDLINE, the Cumulative Index to Nursing and Allied Health Literature (CINAHL), Web of Science, and the Cochrane Library (date last searched 24 December 2012). Combinations of search terms were used related to speaking up (speak* up, speak* out, assertive*), inter-professional relations (inter-professional relations, physician-nurse relations), health personnel (health personnel, patient care team, nursing-supervisory, attitude of health personnel, professional role, professional practice), and patient safety (risk management, safety, medical errors, malpractice, professional misconduct, quality of health care, outcome and process assessment, program evaluation, quality assurance consumer satisfaction, physician’s practice patterns, nurse’s practice patterns, practice management). Our search terms were determined from candidate keywords such as ‘voice’ and ‘challenge’. Speaking-up behaviour can be described using a variety of words; therefore, we adhered to our previous search history to ensure specificity and to improve sensitivity (Additional file 1). The Medical Subject Headings were used where available. The literature searches were conducted with the assistance of experts in library science.

Moreover, hand searches were also conducted of relevant journals on patient safety and organisational research (*Journal of Patient Safety, The Joint Commission Journal on Quality and Patient Safety, BMJ Quality & Safety, Journal of Quality Management in Health Care, Journal of Nursing Management,* and *Journal of Organisational Behaviour*). Furthermore, the referenced articles in each of the selected publications were examined, and the abstracts of relevant congresses were screened. In addition, we discussed team communication with several patient safety experts and asked them to refer us to relevant speaking-up studies.

### Selection of articles

An article was selected only if it fulfilled the following criteria: (1) the subjects of the study were physicians, medical residents, fellows, and/or nurses and (2) the article described the speaking-up behaviour, as well as its barriers, of hospital-based health care professionals within their teams or evaluated the relationship between speaking-up behaviour and patient safety. An article was excluded if it focused on incident-reporting behaviour or communication between health care professionals and patients/their families (e.g. open disclosure to patients) or described only training programs or communication strategies (e.g. communication or speaking-up tools).

### Data extraction

At least two reviewers (AO, Research Assistant) independently reviewed the titles and abstracts of citations generated by the search to assess their eligibility for further review based on the selection criteria and chose relevant articles for possible inclusion. Cohen’s kappa was calculated to assess the degree of agreement between reviewers. The reviewers, supported by the other authors (BB, CW), then reviewed all of the selected articles and decided which to include in this study. The standard Best Evidence in Medical Education coding sheet [14] was modified to focus on relevant parameters (e.g. country, subject, purpose of speaking up, measurement, study design) and used to abstract the information.

This review prioritised articles that appeared to be relevant rather than particular study types or articles that met particular methodological standards [15]. We included a wide variety of articles, including both quantitative and qualitative studies. Therefore, we used the following criteria to assess primary study quality: (1) the aims and objectives of the research were clearly stated, (2) the researchers design was clearly specified and appropriate for the aims and objectives of the research, (3) the researchers provided a clear account of the process by which their findings were reproduced, (4) the researchers displayed enough data to support their interpretations and conclusions, and (5) the method of analysis was appropriate and adequately executed [15].

### Data synthesis

To assess the effectiveness of speaking up for patient safety and the effectiveness of training, two reviewers (AO, Research assistant) independently abstracted the reported outcomes. These outcomes were heterogeneous; therefore, meta-analyses were not conducted. We summarised these results qualitatively. To develop the model of speaking-up behaviour, we began with detailed inspection of the articles, gradually identifying recurring themes, and then generated themes that helped to explain the speaking-up behaviour described in the literature [15]. At the stage of data abstraction, two reviewers (AO, Research Assistant) independently abstracted information (e.g. the influencing factors) and discussed the studies to determine consensus regarding the identification and coding of themes. The identified themes of factors influencing speaking up were integrated into the model of employee’s voicing behaviour [11].

All data included in this report were previously published and publicly available. Hence, our study did not require submission to the local institutional review board for ethical approval.

## Results

### Search results and article overview

**Figure 1:**
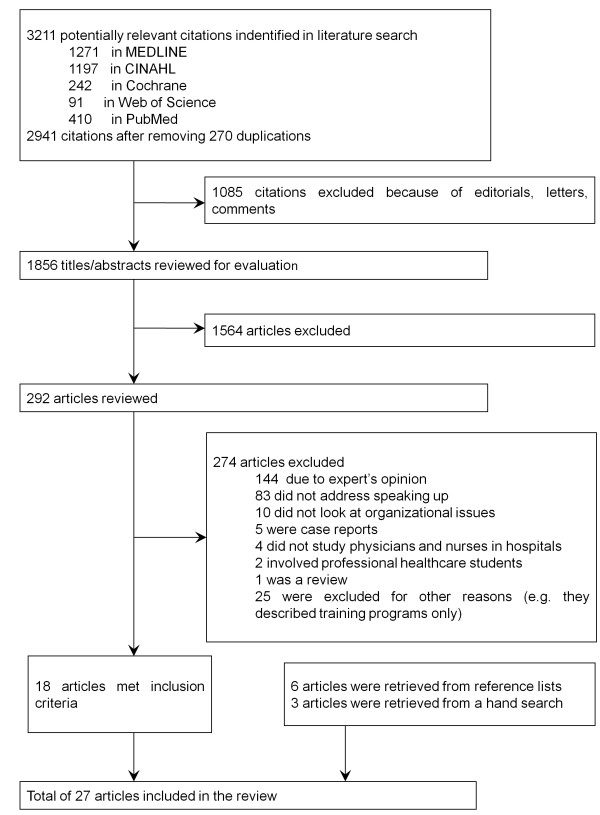
Study selection process.

The initial literature search identified 3,211 citations (Figure 1). Most of the 1,564 excluded articles were based solely on experts’ opinions or commentaries or did not examine speaking-up behaviour in health care teams. In total, 292 articles were filtered for detailed review to determine whether they met the inclusion criteria. Following a title and abstract review by two researchers (AO, Research Assistant), the value of Cohen’s kappa was calculated to be 0.64. A total of 18 articles were found to meet the inclusion criteria; 6 other articles were retrieved from the reference lists, and 3 more were acquired through hand searches. Thus, a total of 26 studies in 27 articles were identified; 7 articles were published in 2012, 2 or 3 were published between 2006 and 2011 each year, and 3 were published before 2006 [3,5,16-40]. More than half of the selected studies (19, 73%) came from the U.S., and of the remaining 7, 3 came from the U.K. (12%). One study was conducted in two countries (the U.S. and Japan) [32]. Most of the selected studies employed interviews and/or surveys, and 8 of the 26 (31%) identified studies described the speaking-up behaviour of physicians. Of the remaining 18 studies, 8 (31%) described the speaking-up behaviour of nurses, and 10 (38%) described the speaking-up behaviour of both physicians and nurses. In all of the included studies, aim, study process, and analysis method were described. Study designs for research purposes were generally selected appropriately. Most of the studies provided sufficient data to support the conclusions, but some provided limited data (Additional file 2).

### Effectiveness of speaking up for patient safety

A few studies directly addressed the relationship between speaking-up behaviour and patient safety outcomes. Among them, three studies [18-20] investigating the pattern of communication failures indicated that, in the case of hesitancy to speak up, insufficient information transfer from residents/nurses to senior physicians could contribute to actual communication errors and/or adverse events. Kolbe et al. demonstrated that nurses’ level of speaking up was a predictor of technical team performance (R_2_ = 0.18, p = 0.17) [16]. Studies that investigated health care professionals’ experiences of speaking up reported that they hesitated to speak up even when they were aware of patient safety risks [33,34,38-40]. Another study reported that 74–78% of residents and attending physicians recalled an incident in which the resident spoke up to prevent an adverse event [27]. All of these studies supported the notion that health care professionals voicing their concerns can be a good opportunity to prevent an adverse event. Churchman and Doherty reported that nurses questioned doctors’ practices only under specific circumstances (e.g. when hospital policies supported the nurse’s position) [34]. Raising concerns was perceived as a high-risk, low-benefit action for nurses [37]. These studies also suggested that by keeping silent, we miss the opportunity to prevent an adverse event and improve patient safety. On the other hand, Jeffs et al. reported that collective vigilance (e.g. the process by which health care professionals would pick up on potentially harmful errors made by another clinician) can potentially create risk by eroding individual professional accountability through reliance on other team members to catch and correct their errors [17]; their study included a limited number of participants from each speciality (e.g. three physicians and one technician). This phenomenon should be evaluated in further study.

### Effectiveness of speaking-up training

Two of the included studies illustrated that the speaking-up behaviour of interns and residents improved after intervention [21,22]; three others reported that, after intervention, the number of participants who felt able to speak up in a clinical setting was increased [24-26]. Stevens et al. reported in their case study that, following team training, communication was enhanced by addressing team members by their names and paying more attention to ‘closing the loop’ in verbal communication, but the amount of data presented by the authors was limited [25].

### Factors influencing speaking up

**Figure 2:**
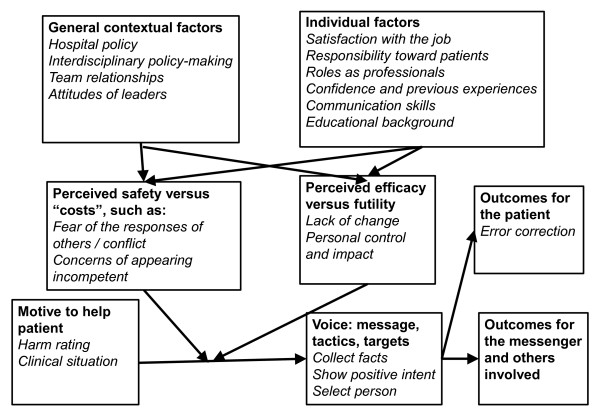
**Model of health care professionals’ speaking up.** Bold shows the framework of Morrison’s model of employee voice. Italic shows identified speaking-up factors

Previous studies have shown that many factors can have an effect on the speaking-up behaviour of health care professionals. These influencing factors could be assigned to the following categories: motivation and clinical context, general contextual factors, individual factors, the perceived safety of speaking up, and the perceived efficacy of speaking up (Figure 2).

Most of the articles that explored the factors influencing speaking-up behaviour used the exploratory approach to find the barriers and promoters of speaking up. All studies described their aim, study design, and results with sufficient data. A few studies investigated the relationship between these factors and speaking-up behaviour [5,29-31].

#### ***Motivation and clinical context***

Perception of a risk for patient or organisation is a prerequisite for speaking up. In one exploratory study, physicians rated potential harm in common clinical scenarios lower than nurses did, and *this harm rating* could also be one of the predictors of speaking up [5]. Also, c*larity or, in contrast, ambiguity of the clinical situation* is an important predictor of the decision to speak up [36,39]. Clarity of the clinical context can be a powerful contributor to the confidence and speaking-up behaviour of health care professionals.

#### ***General contextual factors***

*Strong and visible hospital administrative support* has been shown to enhance the speaking-up behaviour of health care professionals [28,34]. It has been observed that nurses tend to voice their concerns when hospital policies openly support their position [34]. Furthermore, nurses have been shown to want more collegial practice environments in which health care professionals would have more opportunities for interaction, colleagues would treat each other with kindness and consideration, and the ‘different but equal’ contributions of nurses and physicians would be respected (*interdisciplinary policy-making*) [28]. On the other hand, perceived pressure from the nursing team has also been found to have an adverse impact on speaking up for junior physicians [35]. The so-called ‘power nurses’ place those junior physicians, who rely on their assistance, in a vulnerable position; the physicians feel uncomfortable and hesitant at refusing the nurses’ requests, even when they strongly disagree. Moreover, several studies report that *teamwork and a person’s relationships* with other team members influence speaking-up behaviour [3,19,20,27-30,32,34-36]. In particular, *the attitude of a senior or team leader* can have a strong impact on speaking-up behaviour [3,27,30,32]. Coaching by team leaders helps team members to learn from problems and errors [3].

#### ***Individual factors***

It has been found that persons who positively voice their concerns are generally *more satisfied with their workplace* and exhibit more discretionary efforts to speak up [29,39]. Having *a sense of responsibility toward patients* can also have some effect on speaking-up behaviour [34,36,39]. Those who voice their opinions or concerns feel that they create a safer environment for others. The degree of *identification with their roles as physicians or professionals* has been shown to be one of the factors influencing speaking-up behaviour by health care professionals [5,19,38,39]. Perception of a lack of sufficient knowledge is a barrier to speaking up, as health care professionals tend to hesitate to speak up when they feel they are not adequately informed. *A feeling of confidence and previous favourable experiences of speaking up* can enhance such behaviour [5,35,36,38,39].

One study illustrated that *health care professionals’ communication skills*, such as the ability to use assertive and critical language, have an influence on self-confidence and speaking-up behaviour [40].

Furthermore, *the educational background* is also important in understanding a nurse’s speaking-up behaviour [28].

#### ***Perceived safety of speaking up***

Some studies also illustrated that a perceived response from the addressed person (e.g. *fear of reprisal*, *concerns of appearing incompetent*) is an important factor controlling speaking up for both medical and nursing professionals [3,20,28,32,34,37]. Health care professionals were also concerned that voicing their concern could lead to *conflicts* within the health care team [3,32,34].

#### ***Perceived efficacy of speaking up***

*Prediction that nothing will be done* about raised concerns inhibits health care professionals from voicing their concerns [33,37]. *Personal control* (e.g. perceptions of autonomy and *impact* at work) has been found to positively affect the speaking-up behaviour of nurses [29].

#### ***Tactics and targets***

Some nurses *collected facts* as much as possible, ran pilot tests, and worked behind the scenes when the issues were not urgent [40]. They explained their *positive intent*—‘how they wanted to help the caregivers as well as the patient’—while avoiding telling negative stories or making accusations [40]. Nurses sometimes avoided voicing their concern directly to the addressed person, instead telling another person, such as a nurse manager (*selecting person*) [40].

## Discussion

Health care professionals are expected to speak up about their concerns before a critical event reaches a patient to provide a chance to correct the plan or intervention. There have been some studies investigating the relationship between the speaking-up behaviour of health care professionals and patient safety outcomes. They indicate that hesitancy to speak up can be an important contributing factor in communication errors and/or adverse events [18-20]. Most medical and nursing professionals, irrespective of their position and specialty, have some experience of hesitating in voicing their concerns over patient safety risks, even when they are aware of the hazards and immorality of not speaking up [5,27,33-35,38-40]. These studies indicate that, if health care professionals voice their concerns, it may provide the opportunity to recover from errors and avoid adverse consequences, even if there are some biases (e.g. people were likely doing what they were doing because they thought they were right, given their understanding and the pressure of the situation [41]). It is difficult to observe speaking-up behaviour in the clinical setting and to evaluate its effectiveness. Organisational research has illustrated the importance of the voluntary sharing of ideas and information for organisational learning and improvement [3,11,29]. Collecting the cases of speaking up and its outcomes, including the impact on team members, can be an important first step to understanding the consequences of speaking up. Speaking up may affect not only the patient but also the messengers themselves, other team members, and/or the organisation. In this review, we did not focus on these latter issues, and further research is needed to pay attention to how they should be addressed to enhance speaking-up behaviour.

Where training programs have been introduced in order to improve health care professionals’ speaking-up behaviour, there is no strong direct evidence that coaching in speaking up improves patient safety. However, Kolbe et al. demonstrated that a nurse’s level of speaking up is a predictor of technical team performance [16], and appropriate training has been shown to have a positive influence on the speaking-up attitudes [23-25] and behaviour of health care professionals in a simulated setting [21,22]. This provides a rather strong case for health care professionals to undergo training in communication skills (e.g. the use of critical language, assertion, and standardized communication tools) to obtain the know-how to alert team members to unsafe situations [4,42]. The model of speaking-up behaviour helps trainers to design programs that will lead to more effective and sustainable behavioural changes and safety improvement outcomes.

From the literature, we identified various factors that influence speaking up by health care professionals. We integrated these factors into Morrison’s model of employee voice [11] as follows: (1) motivation to speak up to help the patient, such as the perceived risk for patients [5], and the ambiguity or clarity of the clinical situation [36,39]; (2) contextual factors, such as hospital administrative support [28,34], interdisciplinary policy-making[28], team work and a person’s relationship with other team members [3,19,20,27,28,30,34-36], and attitude of leaders/superiors [3,27,31,32]; (3) individual factors, such as satisfaction with the job [29,39], a sense of responsibility toward patients [34,36,39], responsibility as professionals [5,19,38,39], confidence based on experience [5,29,35,36,38,39], communication skills [3,40], and educational background [28]; (4) the perceived safety of speaking up, such as fear of the responses of others and conflict [3,28,32,34,37] and concerns over appearing incompetent [20]; (5) the perceived efficacy of speaking up, such as lack of changes [33,37] or the personal control of the issues [29]; and (6) tactics and targets such as collecting facts, showing positive intent, and selecting the person who will be spoken up to [40]. The model is comprehensive and gives us an overview that helps us to understand why health care professionals do or do not voice their concerns for patient safety. For example, many studies in this review emphasised the importance of team relationships or leaders’ attitudes for speaking up. Thus, for instance, leaders’ inclusiveness can increase a feeling of safety and efficacy of speaking up. However, a recent study found that the perceived behaviour of actual leaders was only modestly correlated with speaking up against them [43]. The authors, therefore, concluded that an employee’s silence is influenced as much by his or her own cognitive frameworks as by a current boss’s behaviour or by organisational factors [43]. Speaking-up behaviour might, accordingly, not be directly influenced by perceived team relationships and leaders’ attitude so much as indirectly by the perception of efficacy or safety of speaking up.

Factors influencing speaking-up behaviour will depend upon the organisation. Voicing in another organisation may be aimed at defending the interests of the organisation, client, third party, speaker, or a combination of these. The motivation to speak up for patient safety is primarily intended to prevent avoidable injury to the client. On the other hand, there is a potential to learn further from other sectors. For instance, no study in a health care setting focuses on work-group size and structure, while these are reported to influence employees’ voicing behaviour in other organisations [11]. This may be a topic for future research.

This review has its own limitations. First, we developed the model of speaking-up behaviour by health care professionals based on previous studies in the health care setting. Further study based on this theoretical framework is required to investigate the relative importance of the different factors influencing speaking-up behaviour in various health care settings and the validity of the model. Second, in this review, similarities were found between factors influencing the speaking-up behaviour of junior physicians and factors influencing that of nurses, but the impact of these factors may differ between these groups. In addition, most selected studies were conducted in Western countries, so the factors influencing speaking up may be different in other countries. Further research is necessary to determine the impact of each controlling factor on the speaking-up behaviour of different caregivers with different cultural backgrounds. Finally, due to the variation in language used to express the term ‘speaking up’ in the literature, we used several keywords in searching for articles. Despite using combinations of search terms and a thesaurus, we were unable to further improve upon either the sensitivity or specificity of our literature search; some articles may, therefore, have been overlooked. To compensate for this, we consulted several experts and checked relevant journals to find related articles. Despite these limitations, this review helps us to understand how health care professionals think about voicing their concerns for patient safety.

## Conclusion

Hesitancy to speak up is one of the factors that may contribute to communication errors and/or adverse events. Many junior physicians and nurses have experience hesitating to voice their concerns over patient safety, even when they are aware of the risks and the shortcomings of such omissions. If health care professionals candidly speak up about their concerns for patient safety, this may provide a good opportunity to avoid errors or to recover from them. Many factors can influence the speaking-up behaviour of health care professionals. The presented model can help to provide an understanding of the complexity of these controlling factors. Our model can be useful for trainers to develop training programs and also for trainee’s self-reflection.

## Competing interest

The authors declare that they have no competing interests.

## Authors’ contributions

AO had full access to all of the data in the study and takes full responsibility for the integrity of the data and the accuracy of data analysis. AO, CW, and BB designed the study and analysed data. AO drafted the manuscript. CW and BB supervised the study and provided comments on subsequent versions of the manuscript. All authors read and approved the final manuscript.

## Pre-publication history

The pre-publication history for this paper can be accessed here:

http://www.biomedcentral.com/1472-6963/14/61/prepub

## Supplementary Material

Additional file 1 Search strategy for MEDLINE. Click here for file

Additional file 2 The selected study and its quality assessment. Click here for file
