# Supplementary material for: Speaking up for patient safety by hospital-based health care professionals: a literature review
Source: BMC Health Serv Res. 2014 Feb 8;14:61. doi: 10.1186/1472-6963-14-61 (PMC4016383; doi:10.1186/1472-6963-14-61)
Supplement: Additional file 1 — Search strategy for MEDLINE. [file 1472-6963-14-61-S1.pdf]

Appendix 1. Search strategy for MEDLINE

Database: Ovid MEDLINE(R) In-Process & Other Non-Indexed Citations <December 24, 2012>, Ovid MEDLINE(R) 1946 to Present with Daily Update, Ovid OLDMEDLINE(R) <1946 to 1965>
Search Strategy:
----

1 Assertiveness/ (1556)
2 assertiveness.tw. (1197)
3 "speak* up".tw. (316)
4 or/1-3 (2561)
5 exp *Health Personnel/ (249028)
6 exp *Patient Care Team/ (20745)
7 *Nursing, Supervisory/ (5431)
8 *"Attitude of Health Personnel"/ (42634)
9 exp *Professional Role/ (30967)
10 *Professional Practice/ (7690)
11 or/5-10 (321860)
12 4 and 11 (577)
13 *Interprofessional Relations/ (16394)
14 *Physician-Nurse Relations/ (973)
15 or/13-14 (17339)
16 exp *Safety/ (18232)
17 exp *Risk Management/ (34376)
18 exp *Medical Errors/ (22441)
19 exp *Malpractice/ (20327)
20 *Professional Misconduct/ (1745)
21 *"Quality of Health Care"/ (26462)
22 exp *"Outcome and Process Assessment (Health Care)"/ (32214)
23 *Program Evaluation/ (6664)
24 *Quality Assurance, Health Care/ (26651)
25 exp *Consumer Satisfaction/ (27093)
26 *Physician's Practice Patterns/ (22237)
27 *Nurse's Practice Patterns/ (475)
28 *Practice Management, Medical/ (6831)
29 *Practice Management/ (736)

30 or/16-29 (218106)
31 15 and 30 (1045)
32 or/12,31 (1617)
33 exp *Persons/ (785527)
34 exp *Patients/ (31211)
35 11 not 34 (318472)
36 33 not 35 (531406)
37 exp *Family/ (114504)
38 exp *Professional-Patient Relations/ (52655)
39 *Professional-Family Relations/ (4687)
40 *"Patient Acceptance of Health Care"/ (15702)
41 exp *Patient Compliance/ (20570)
42 *Patient Advocacy/ (11872)
43 exp *Education, Nonprofessional/ (108922)
44 exp *Consumer Participation/ (15911)
45 *Consumer Advocacy/ (1499)
46 exp *Patient Rights/ (32989)
47 *Disclosure/ (4113)
48 *Truth Disclosure/ (6142)
49 *Hospital-Patient Relations/ (1074)
50 *Sexual Harassment/ (887)
51 exp *Violence/ (49874)
52 exp *Emotions/ (82084)
53 or/34,36-52 (905120)
54 32 not 53 (1386)
55 limit 54 to (dutch or english or japanese) (1314)
56 limit 55 to (comment or editorial or introductory journal article or letter or news or newspaper article or overall) (167)
57 55 not 56 (1147)
58 remove duplicates from 57 (1110)
