# Supplementary material for: Speaking up for patient safety by hospital-based health care professionals: a literature review
Source: BMC Health Serv Res. 2014 Feb 8;14:61. doi: 10.1186/1472-6963-14-61 (PMC4016383; doi:10.1186/1472-6963-14-61)
Supplement: Additional file 2 — The selected study and its quality assessment. [file 1472-6963-14-61-S2.pdf]

Appendix 2. The selected studies and **its quality assessment**

**Author (Year)**	**Study aim**	**Method**	**Sample size and description**	**Main results**<br>1. Relationship between speaking up behaviour and safety<br>2. Speaking up behaviour<br>3. Influencing factors	**Quality assessment ** **
**Kolbe et al. (2012)[16]**	To test the relationship between speaking up and technical team performance.	Prospective observational study, linear regression analysis.	2-person ad hoc anaesthesia teams (31 nurses, 31 residents) in Switzerland.	1. A trend indicated that nurses' previous levels of speaking up, not those of their respective colleagues, predicted later speaking up behaviour. Nurses' levels of speaking up are a predictor of technical team performance ($R2=0.18$, $p=0.17$). Team members reacted immediately to speaking up.	1. Yes<br>2. Yes<br>3. Yes<br>4. Yes<br>5. Yes
**Jeffs et al. (2012)[17]**	To explore how different health care professions experience and respond to near misses.	Interview, content analysis.	24 clinicians in a large teaching hospital in Canada.	1. Collective vigilance can potentially create risk by eroding individual professional accountability through reliance on other team members to catch and correct their errors.	1. Yes<br>2. Yes<br>3. Yes<br>4. Weak<br>5. Yes
**Rabøl et al. (2011)[18]**	To review root cause analysis reports for descriptions of verbal communication.	Case study, content analysis.	84 root cause analyses from 6 Danish hospitals.	1. Hesitance in speaking up contributed to 10 communication errors (23%) out of a total of 44.	1. Yes<br>2. Yes<br>3. Yes<br>4. Yes<br>5. Yes
**Greenberg et al. (2007)[19]**	To identify patterns of communication breakdown.	Malpractice claim review, content analysis.	444 surgical malpractice claims from 4 US liability insurers.	1. All nine of the resident-to-attending communication breakdowns related to information not being transmitted from the resident to the attending.<br>3. Status asymmetry, ambiguity about role and responsibilities.	1. Yes<br>2. Yes<br>3. Yes<br>4. Yes<br>5. Yes
**Sutcliffe et al. (2004)[20]**	To describe how communication failures contribute to medical mishaps.	Interview, grounded theory approach.	26 residents at a 600-bed US teaching hospital.	1. Aspects of communication, including hesitance in speaking up, and patient management were the two most commonly cited contributing factors.<br>3. Hierarchy, concerns about appearing incompetent in front of those with more power.	1. Yes<br>2. Yes<br>3. Yes<br>4. Yes<br>5. Yes

**The effect of training and intervention**					
**Thomas et al. (2007)[21]**	To evaluate the effect of a team training program on patient safety.	Observational cohort study with control group, Mann Wnitney rank test.	51 interns from the US.	3. Training: the interns in the team training group exhibited more frequent team behaviours than interns in the control group (number of episodes per minute (95%CI): assertion 1.80 (1.21, 2.25) vs. 0.64 (0.26, 0.91) (p<0.008)).	1. Yes<br>2. Yes<br>3. Yes<br>4. Yes<br>5. Yes
**Pian-Smith et al. (2009)[22]**	To evaluate the effect of educational intervention on patient safety.	Observational before and after study, Wilcoxon signed rank test.	40 residents from the US.	3. Training: overall use of the two-challenge rule (speaking up) and advocacy-inquiry increased after implementation debriefing.	1. Yes<br>2. Yes<br>3. Yes<br>4. Yes<br>5. Yes
**Sayre et al. (2012)[23]**	To investigate whether in-service training could lead nurses to speak up, thereby enhancing perception of collaboration.	Quasi-experimental survey, regression analysis.	Nurses recruited from two similar acute care hospitals in the US.	3. After the intervention, there was a statistically significant difference for the intervention group baseline and post-test speaking up measurement scores (P<0.001), and CPS scores (P<0.000). Correlation among the baseline mean scores of the speaking up measure and CPS for the intervention group indicated a strong relationship (r=0.64). The post-intervention scores maintained a moderately strong relationship (r=0.47).	1. Yes<br>2. Yes<br>3. Yes<br>4. Yes<br>5. Yes
**Sayre et al. (2012)[24]**	To evaluate the effect of educational intervention on patient safety.	Quasi-experimental survey, regression analysis.	145 nurses from two 300-bed acute care hospitals in the US.	3. Training: a significant difference in self-reported speaking up behaviours and scores in the intervention group (P<0.001).	1.Yes<br>2. Yes<br>3. Yes<br>4. Yes<br>5. Yes
**Stevens et al. (2012)[25]**	To develop a comprehensive program to monitor the performance of experienced cardiac surgical teams.	Survey before and after training (pilot study), mixed effect ordinal logistic regression analysis.	Working cardiac surgery operating room including surgeon, anaesthesiologist, nurse etc. in Canada.	3. After simulated training, about half of those interviewed answered that they were able to speak up more readily, communicate more clearly by addressing team members by their names, and were paying more attention to “closing the loop” in verbal communications.	1. Yes<br>2. Yes<br>3. Yes<br>4. Weak<br>5. Yes
**Johnson et al. (2012)[26]**	To implement a team training program.	Case study, descriptive analysis.	Clinicians, including physicians and nurses, in a preoperative division of the Lehigh Valley Health Network in the US.	3. Post-course evaluations showed that the majority of respondents believed they were better able to question the decisions or actions of someone with more authority.	1. Yes<br>2. Yes<br>3. Yes<br>4. Weak<br>5. Yes

**The influencing factors**					
**Lyndon et al. (2012)[5]**	To explore factors that may predict whether clinicians speak up in the face of safety concerns.	Survey, multiple linear regression analysis.	125 clinicians from two US labour & delivery units.	2. Some participants (12%) indicated they were unlikely to speak up, despite perceiving a high potential for harm in certain situations.<br>3. A higher perception of harm, respondent role, specialty experience and site predicted the likelihood of speaking up when controlling for bravery and assertiveness.	1. Yes<br>2. Yes<br>3. Yes<br>4. Yes<br>5. Yes
**Belyansky et al. (2011)[27]**	To examine the factors that influence surgical trainees in expressing their opinions.	Survey, descriptive analysis.	38 residents and 23 attending in a US hospital.	2. 40% of residents and 47% of attending indicated that they were aware of an intraoperative incident where the resident knew something was wrong but did not speak up. 74-78% of residents and attending recalled an incident where the resident spoke up and prevented an adverse event.<br>3. Attending personality, interpersonal relationships.	1. Yes<br>2. Yes<br>3. Yes<br>4. Yes<br>5. Yes
**Simpson et al. (2009)[28]**	To describe how nurses would respond to common clinical contexts involving disagreements.	Survey, descriptive analysis.	133 nurses at a hospital in the US.	3. Hierarchy, fear of and actual intimidation by physician colleagues, lack of administrative support, mutual respect, interdisciplinary policy-making, and education.	1. Yes<br>2. Yes<br>3. Yes<br>4. Yes<br>5. Yes
**Tangirala et al. (2008)[29]**	To examine the effects of procedural justice climate on employee silence.	Survey, hierarchical linear modeling.	606 nurses nested within 30 workgroups from a large Midwestern hospital in the US.	3. Procedural justice climate (consensual group-level cognitions of evaluating the fairness of organizational authorities).	1. Yes<br>2. Yes<br>3. Yes<br>4. Yes<br>5. Yes
**Tangirala et al. (2008)[30]**	To examine the relationship between personal control and voice.	Survey, hierarchical linear modeling.	586 nurses and their nurse managers from a large Midwestern hospital in the US.	3. Personal control, organizational identification.	1. Yes<br>2. Yes<br>3. Yes<br>4. Yes<br>5. Yes
**Nembhard et al. (2006)[31]**	To examine the relationship between status and psychological safety (feeling secure enough to speak up about issues or ideas).	Survey, general linear model.	1,440 clinicians at 23 neonatal intensive care units in the US.	3. Training leaders to be inclusive (words and deeds exhibited by leaders that invite and appreciate others' contributions) to foster psychological safety (feeling secure enough to speak up about issues or ideas).	1. Yes<br>2. Yes<br>3. Yes<br>4. Yes<br>5. Yes
**Kobayashi et al. (2006)[32]**	To compare residents' attitudes to speaking up in the US and Japan.	Survey, descriptive analysis.	175 US residents, 65 Japanese residents from academic medical centres.	3. Relationship with superiors and their perceived responses.	1. Yes<br>2. Yes<br>3. Yes<br>4. Yes<br>5. Yes

**Rutherford et al. (2012)[33]**	To investigate the non-technical skills involved in the effective teamwork of anaesthetic assistants.	Interview, content analysis.	22 anaesthetic assistants and consultant anaesthetists in the UK.	2. 26% (N=5) indicated that they would not speak up.<br>3. Just because someone says that they will speak up does not always mean that they will in practice.	1. Yes<br>2. Yes<br>3. Yes<br>4. Yes<br>5. Yes
**Maxfield et al. (2010) The 'silent treatment' study*[40]**	To quantify decisions not to speak up.	Survey, descriptive analysis.	6,618 nurses and 1,001 nurse managers in the US.	2. Caregivers are often unable to speak up and resolve their concerns about dangerous shortcuts, incompetence, and disrespect.<br>3. Positive intent (e.g. protection of the other person), the ability to be assertive and to use critical language. Working behind the scene to collect facts, working showing positive intent, and selecting the person who is spoken up.	1. Yes<br>2. Yes<br>3. Yes<br>4. Yes<br>5. Yes
**Churchman et al. (2010)[34]**	To explore the extent to which nurses are willing to challenge doctors' practices.	Interview, thematic approach.	12 nurses from an acute hospital in the UK.	2. Nurses questioned doctors' practices only under specific circumstances (e.g. when hospital policies supported her position).<br>3. Patient advocates, hospital policies. Nurses would not challenge doctors if they perceived that this would result in conflict or stress, if they were afraid of the doctor, or if they feared reprisal.	1. Yes<br>2. Yes<br>3. Yes<br>4. Yes<br>5. Yes
**Lewis et al. (2009)[35]**	To explore uncomfortable prescribing decisions, including hesitance to voice concerns about inappropriate prescriptions.	Interview, grounded theory approach.	48 physicians from 4 UK hospitals.	2. Doctors admitted to prescribing to maintain overall team relationships without speaking up, sometimes ignoring hospital regulations and best practice to do so.<br>3. Hierarchy, perceived pressure from other team members, confidence based on knowledge and experience.	1. Yes<br>2. Yes<br>3. Yes<br>4. Yes<br>5. Yes
**Lyndon. (2008)[36]**	To identify the processes affecting nurses', physicians' and certified nurse-midwives' responsibilities for patient safety and treatment.	Interview, grounded theory approach.	12 nurses, 5 physicians, and 2 midwives from two academic prenatal units in the US.	3. Clinical context, interpersonal relationships, hierarchy, perceived level of knowledge, protection of the other person, avoidance of conflict, patient advocacy, fluctuating agency for safety, experience and confidence.	1. Yes<br>2. Yes<br>3. Yes<br>4. Yes<br>5. Yes
**Attree. (2007)[37]**	To explore factors that influence nurses' decisions to raise concerns about standards of practice.	Interview, grounded theory approach.	142 nurses from 3 acute NHS trusts in England.	2. Raising concerns was perceived as a high-risk: low-benefit action.<br>3. Fear of repercussions, retribution, labelling and blame for raising concerns, about which they predicted nothing would be done.	1. Yes<br>2. Yes<br>3. Yes<br>4. Yes<br>5. Yes

**Blatt et al. (2006)[38]**	To explore how medical residents dealt with medical mishaps they witnessed (e.g. whether or not they voiced their concerns).	Interview, grounded theory approach.	26 residents at a 550-bed US teaching hospital.	2. Medical residents were aware of a lapse and had an issue of concern to voice that could have helped mitigate or correct the lapse, but instead they remained silent.<br>3. Stronger identity as physicians, and confidence based on knowledge and experience.	1. Yes<br>2. Yes<br>3. Yes<br>4. Yes<br>5. Yes
**Maxfield et al. (2005) The 'silence kills' study*[39]**	To identify topics of difficulty in speaking up, and to examine their prevalence.	Mixed method; observation and focus group interview, content analysis.	More than 1,700 nurses, physicians, clinical-care staff, and administrators in the US.	2. Fewer than one in ten speak up about all of their concerns (broken rules, mistakes, lack of support, incompetence, poor teamwork, disrespect, and micromanagement).<br>3. Those with a sense of responsibility toward patients, commitment to their unit and hospital, and workplace satisfaction exhibit more discretionary effort in speaking up. Those who feel they lack ability, believe that voicing concerns is "not their job", and have low confidence do not speak up. The clinical context also influences the decision to speak up.	1. Yes<br>2. Yes<br>3. Yes<br>4. Yes<br>5. Yes
**Edmondson. (2003)[3]**	To explore the types of leaders' behaviour that promotes speaking up in the context of in-role behaviour while learning new tasks and coordination routines.	Multiple case study, non-parametric statistical test.	Interviews of 165 OR team members in 16 cardiac-surgery teams in the US.	3. Team leader coaching, a history of one-way communication, or of others not being asked for, or providing, input as barriers, physicians over- or under-reacting to the errors of others, communication with a sense of humility.	1. Yes<br>2. Yes<br>3. Yes<br>4. Yes<br>5. Yes
